# The Anti-Adiposity Mechanisms of Ampelopsin and Vine Tea Extract in High Fat Diet and Alcohol-Induced Fatty Liver Mouse Models

**DOI:** 10.3390/molecules27030607

**Published:** 2022-01-18

**Authors:** Jianbo Wu, Kenchi Miyasaka, Wakana Yamada, Shogo Takeda, Norihito Shimizu, Hiroshi Shimoda

**Affiliations:** Research and Development Division, Oryza Oil and Fat Chemical Co., Ltd., 1 Numata, Kitagata-cho, Ichinomiya 493-8001, Aichi, Japan; fengyun771984@gmail.com (J.W.); kgohedasato@gmail.com (K.M.); kgohedasato3@gmail.com (W.Y.); kgohedasato2@gmail.com (S.T.); oryza12702427@gmail.com (N.S.)

**Keywords:** *Ampelopsis grossedentata*, vine tea, ampelopsin, obesity, fatty liver, abdominal fat

## Abstract

*Ampelopsis grossedentata* (AG) is an ancient medicinal plant that is mainly distributed and used in southwest China. It exerts therapeutic effects, such as antioxidant, anti-diabetic, and anti-inflammatory activities, reductions in blood pressure and cholesterol and hepatoprotective effects. Researchers in China recently reported the anti-obesity effects of AG extract in diet-induced obese mice and rats. To verify these findings, we herein investigated the effects of AG extract and its principal compound, ampelopsin, in high-fat diet (HFD)- and alcohol diet-fed mice, olive oil-loaded mice, and differentiated 3T3-L1 cells. The results obtained showed that AG extract and ampelopsin significantly suppressed increases in the weights of body, livers and abdominal fat and also up-regulated the expression of carnitine palmitoyltransferase 1A in HFD-fed mice. In olive oil-loaded mice, AG extract and ampelopsin significantly attenuated increases in serum triglyceride (TG) levels. In differentiated 3T3-L1 cells, AG extract and ampelopsin promoted TG decomposition, which appeared to be attributed to the expression of hormone-sensitive lipase. In alcohol diet-fed mice, AG extract and ampelopsin reduced serum levels of ethanol, glutamic oxaloacetic transaminase (GOT), and glutamic pyruvic transaminase (GPT) and liver TG. An examination of metabolic enzyme expression patterns revealed that AG extract and ampelopsin mainly enhanced the expression of aldehyde dehydrogenase and suppressed that of cytochrome P450, family 2, subfamily e1. In conclusion, AG extract and ampelopsin suppressed diet-induced intestinal fat accumulation and reduced the risk of fatty liver associated with HFD and alcohol consumption.

## 1. Introduction

The prevalence of obesity, which is becoming a public health issue in advanced nations, has already tripled since 1975 according to the World Health Organization. Approximately 1.9 billion adults worldwide are estimated to be overweight, with 650 million being obese. The number of children who are overweight and becoming obese is also increasing [1].

Obesity is generally attributed to the consumption of a high calorie diet or excessive alcohol and metabolic disorders, and a chronic obese status is associated with a number of complications, including fatty liver, hypertension, hyperlipidemia, impaired glucose tolerance, chronic kidney disease, and cardiovascular disease [1,2]. Obesity increases the risk of developing liver diseases, particularly non-alcoholic fatty liver disease (NAFLD) and alcoholic liver disease (ALD). The spectrum of lesional stages in liver damage ranges from simple steatosis to steatohepatitis, hepatic inflammation, and fibrosis, ultimately progressing to cirrhosis, cancer, and liver failure [2]. In the early stage of obesity, the accumulation of triglycerides (TG) and cholesterol occurs in the liver due to an excessive intake of lipids and dysfunctional lipid metabolism. With the progression of symptoms, treatment strategies for obesity focus on reducing TG absorption, while promoting their decomposition. Furthermore, lifestyle modifications and exercise are important strategies for the treatment of obesity, and a number of medicines, such as orlistat, naltrexone/bupropion, phentermine/topiramate, and sibutramine, have also developed. Orlistat is the mostly commonly used medicine in clinical settings, and has been shown to reduce body weight by inhibiting pancreatic lipases in the mucous membranes of the small intestine, thereby preventing the decomposition of TG and their absorption from the intestines [3,4,5]. However, these medicines generally have a number of side effects, including appetite suppression, headache, and liver injury. Therefore, many dietary supplements have recently been developed for obesity, particularly those that promote weight loss and inhibit lipid absorption. The most well-known supplement is l-carnitine, which is listed in the United States Pharmacopoeia. It has been shown to facilitate the transport of fatty acids into mitochondria for β-oxidation [6]. Another well-known supplement is green tea extract, which has been marketed and consumed to reduce cholesterol and low-density lipoproteins and promote weight loss [7]. Many institutes and researchers have attempted to identify new components and develop supplements that are beneficial for the treatment of obesity with fewer side effects.

*Ampelopsis grossedentata* (AG), also called “vine tea”, is an ancient plant with therapeutic effects that is mainly distributed in the southwest China was investigated in the present study. AG leaf extract and its main component ampelopsin (also known as dihydromyricetin) have been shown to exert many beneficial effects, such as antioxidant, anti-diabetic, and anti-inflammatory activities, reductions in blood pressure and cholesterol, and hepatoprotective effects [8,9]. Wan et al. [10] reported that vine tea extract regulated glucose and lipid metabolism and attenuated the hepatic accumulation of lipids in rats fed a high-fat diet (HFD). Xie et al. [11] demonstrated that the administration of vine tea extract (containing approximately 65% ampelopsin) for 3 months prevented Western diet-induced NAFLD in mice by balancing fatty acid oxidation and lipogenesis through increases in the expression of carnitine palmitoyl transferase (CPT) 1A and cytochrome P450, family 4, subfamily a1. It also improved the gut microbiome, which may be related to fat absorption. Research on the chemical components of AG revealed that it contained high levels of polyphenols, particularly ampelopsin, which exerts many of the same effects as AG extract. Chen et al. [12] reported that ampelopsin promoted glucose and lipid metabolism and exerted anti-inflammatory effects in human NAFLD. Silva et al. [13] also showed that it ameliorated steatosis and reduced the accumulation of TG and injury markers in the livers of mice chronically fed ethanol. The underlying mechanism involved the activation of adenosine 5′-monophosphate-activated protein kinase (AMPK) and its downstream targets, including CPT1A, as well as acetyl CoA carboxylase (ACC). Collectively, these findings suggest that AG is a valuable plant for adjusting lipid metabolism and protecting the liver, which is closely associated with the development of obesity.

We speculated that AG extract and ampelopsin may reduce obesity and we describe herein in vivo and in vitro obesity experiments conducted to verify this hypothesis. We compared the anti-obesity effects of AG extract in vivo with the findings reported by Xie [11] by using a HFD-induced NAFLD mouse model instead of a Western diet-induced NAFLD mouse model. HFD in the present study contained more fat and calories, which induced NAFLD in mice within 2 weeks. We employed two other in vivo models, an ALD mouse model recommended by the National Institutes of Health and an olive oil-loaded mouse model, to investigate the effects of AG extract and ampelopsin. In vitro experiments were also performed. Moreover, we examined the effects of AG extract and ampelopsin on pancreatic lipase activity, lipolysis, in a differentiated 3T3-L1 cell culture system and rat epididymal fat pads. Based on the results obtained, the potential mechanisms of action of AG extract and ampelopsin have been summarized and discussed.

## 2. Results

### 2.1. HPLC Analysis of AG Extract

**Figure 1:**
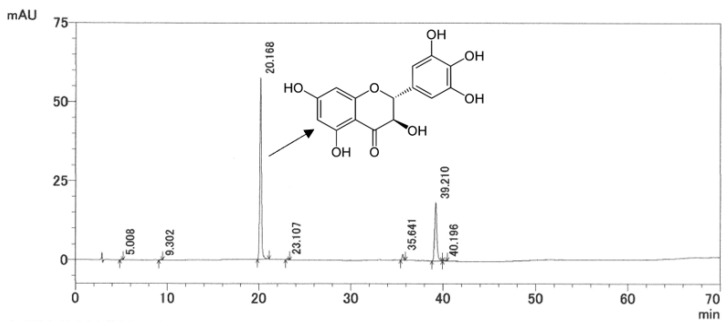
HPLC chromatogram of AG extract.

A HPLC chromatogram of AG extract and the structure of ampelopsin are shown in Figure 1. The content of ampelopsin in AG extract used in the present study was 51.2%.

### 2.2. Effects of AG Extract and Ampelopsin on HFD-Induced NAFLD in Mice

**Figure 2:**
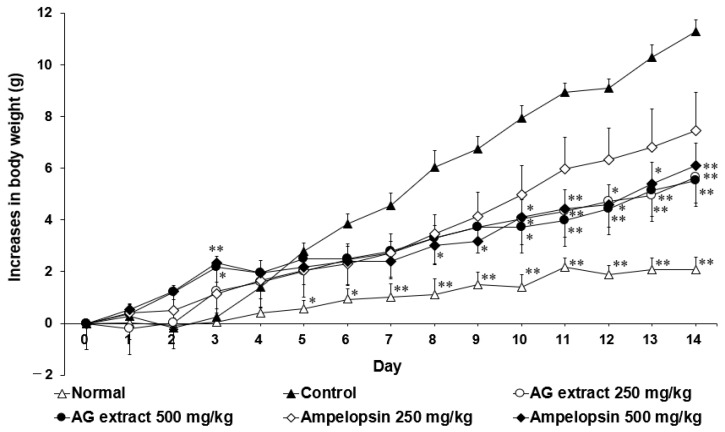
Effects of AG extract and ampelopsin on HFD-induced body weight changes in mice. Each symbol represents the mean ± SE (*n* = 7). Data were assessed by a one-way analysis (ANOVA) of variance followed by Dunnett’s method. Asterisks denote significant differences from the control group at * *p* < 0.05, ** *p* < 0.01.

In comparisons with the control group, the body weights of the groups treated with AG extract (250 and 500 mg/kg) and ampelopsin (500 mg/kg) were markedly lower from day 6 (Figure 2). On day 14, the weights of the liver, epididymal fat, and perirenal fat in the groups treated with AG extract and ampelopsin were also lower (Table 1), particularly those of the liver and epididymal fat. Furthermore, serum TG and total cholesterol (T-Cho) levels were reduced in the groups treated with AG extract and ampelopsin (Table 2), particularly in TG levels those treated with AG extract (250 mg/kg) and ampelopsin (250 mg/kg).

### 2.3. Effects of AG Extract and Ampelopsin on CPT1A Expression in the Liver of HFD-Fed Mice

**Figure 3:**
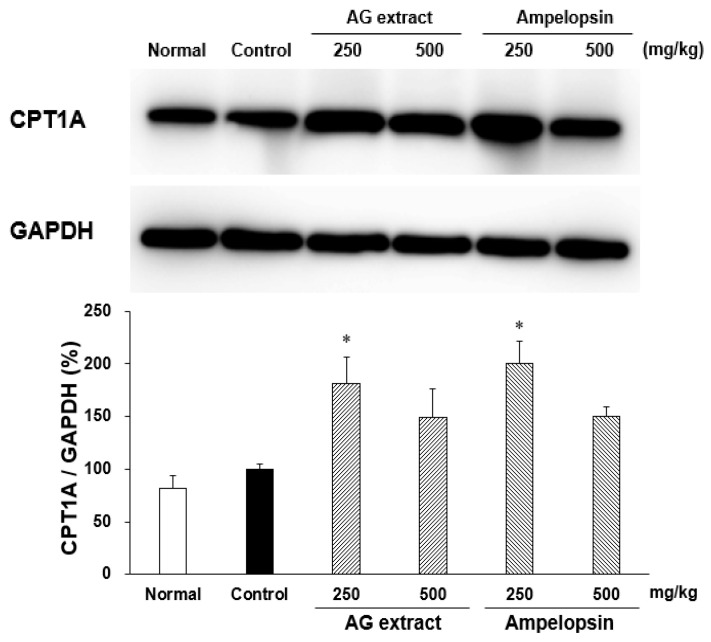
Effects of AG extract and ampelopsin on CPT1A protein expression in the liver of HFD-fed mice. Each column represents the mean ± SE (*n* = 3–4). Data were assessed by a one-way analysis (ANOVA) of variance followed by Dunnett’s method. Asterisks denote significant differences from the control group at * *p* < 0.05.

CPT1A is a key enzyme in carnitine-dependent transport across the mitochondrial inner membrane in the β-oxidation of long-chain fatty acids. In comparisons with the control group, CPT1A expression was higher, particularly in groups treated with AG extract (250 mg/kg) and ampelopsin (250 mg/kg) (Figure 3).

### 2.4. Effects of AG Extract and Ampelopsin on Lipid Absorption in Olive Oil-Loaded Mice

**Figure 4:**
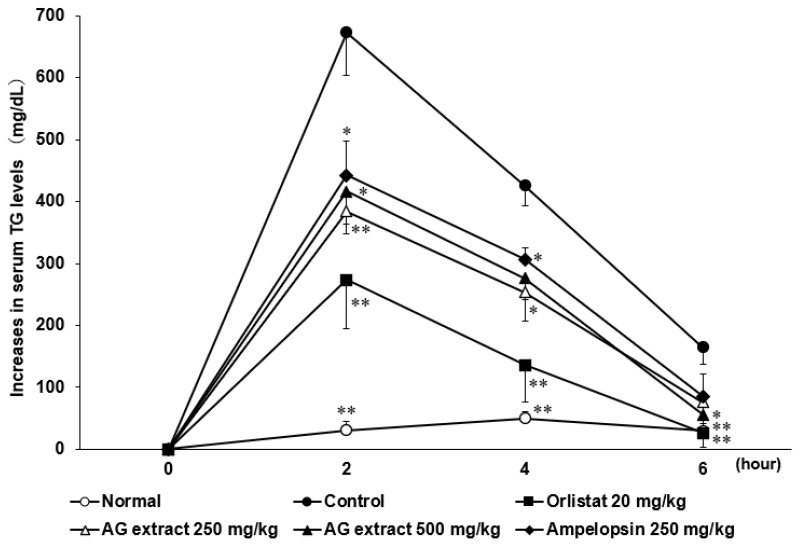
Effects of AG extract and ampelopsin on lipid absorption in olive oil-loaded mice. Each symbol represents the mean ± SE (*n* = 6). Data were assessed by a one-way analysis (ANOVA) of variance followed by Dunnett’s method. Asterisks denote significant differences from the control group at * *p* < 0.05, ** *p* < 0.01.

To confirm the effects of AG extract and ampelopsin on lipid absorption from the intestines, changes in serum TG levels in olive oil-loaded mice were examined. As shown in Figure 4, serum TG levels were higher in the control group than in normal non-loaded mice. Suppressive effects were observed in the groups treated with AG extract and ampelopsin. Compared to the control group, in the groups treated with AG extract (250 and 500 mg/kg), a significant decrease in serum TG levels was observed 2 and 4 h after olive oil loading, while in the group treated with ampelopsin (250 mg/kg), a significant decrease in serum TG levels was noted 2 h after olive oil loading. On the other hand, orlistat (20 mg/kg) significantly inhibited increases in serum TG levels 2, 4, and 6 h after olive oil loading.

### 2.5. Effects of AG Extract and Ampelopsin on Lipolysis in Differentiated 3T3-L1 Adipocytes

**Figure 5:**
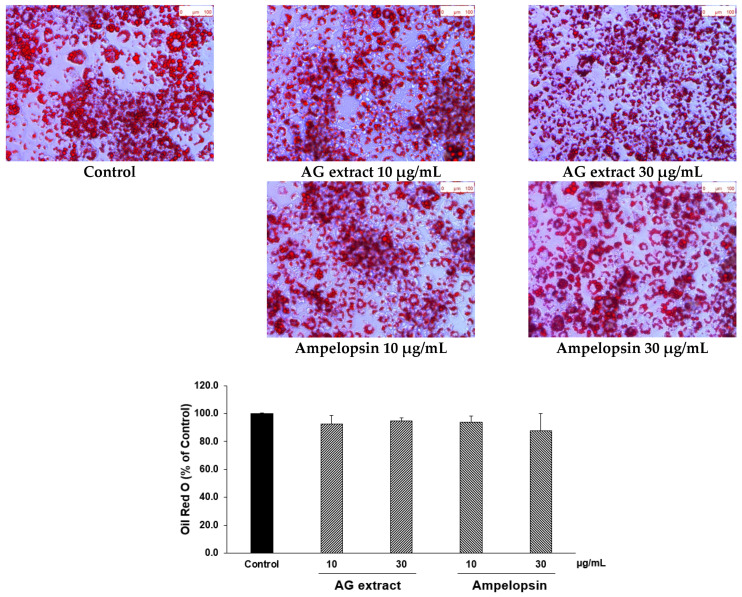
Effects of AG extract and ampelopsin on lipolysis in differentiated 3T3-L1 adipocytes. Each column represents the mean ± SE (*n* = 2–3).

AG extract and ampelopsin did not affect cell viability at concentrations lower than 30 µg/mL (Table 3). In comparisons with the control group, Oil Red O staining revealed that AG extract significantly promoted TG decomposition in 3T3-L1 adipocytes after 48 h, particularly at concentrations of 10 and 30 µg/mL (Table 3 and Figure 5). In concentration of 100 µg/mL, a significant decrease of cell viability was observed in both AG extract and ampelopsin group. We guessed that maybe 100 µg/mL of both samples might have a slight toxicity to 3T3-L1 cells, so we considered that 30 µg/mL was better than 100 µg/mL to evaluate the lipolytic effect. Also, dose dependent inhibition by AG extract was seen from 1 to 10 µg/mL and not seen in ampelopsin group, therefore other compounds might be involved in lipolytic activity of AG extract and lipolytic activity might be blocked by the cytotoxicity of ampelopsin at 30 µg/mL.

### 2.6. Effects of AG Extract and Ampelopsin on Free Fatty Acid (FFA) and Glycerol Release from the Rat Epididymal Fat Pad

In comparisons with the control group, the contents of FFA and glycerol released from the rat epididymal fat pad were significantly increased in the groups treated with AG extract and ampelopsin (Table 4), which suggested that AG extract and ampelopsin promoted fat decomposition. Norepinephrine (NA), which binds to β_3_ adrenergic receptors and promotes FFA and glycerol release from adipocytes, also appeared to promote fat decomposition.

### 2.7. Effects of AG Extract and Ampelopsin on Pancreatic Lipase Activity

Pancreatic lipase, an enzyme secreted from the pancreas, is the primary enzyme hydrolyzing dietary fat in the digestive tract. In comparisons with the control group, AG extract (30 and 100 µg/mL) and ampelopsin (10–100 µg/mL) significantly inhibited the activity of pancreatic lipase in dose dependent manner (Table 5). Maximum inhibition was observed at 100 µg/mL. Orlistat also exerted strong inhibitory effects on the activity of pancreatic lipase.

### 2.8. Effects of AG Extract and Ampelopsin on Alcohol Diet-Fed Mice

**Figure 6:**
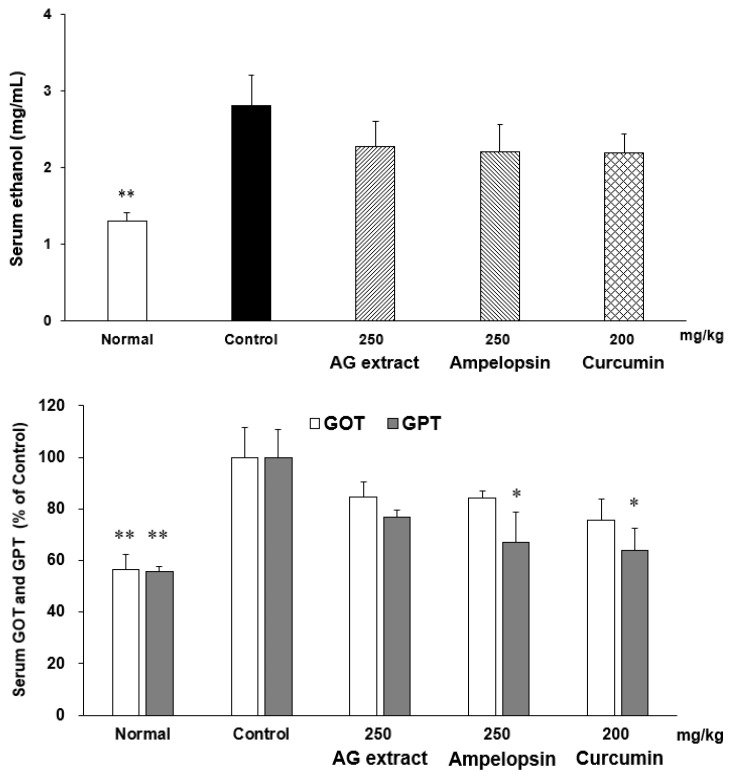
Effects of AG extract and ampelopsin on serum ethanol, GOT and GPT in alcohol diet-fed mice. Each column represents the mean ± SE (*n* = 5–6). Data were assessed by a one-way analysis (ANOVA) of variance followed by Dunnett’s method. Asterisks denote significant differences from the control group at * *p* < 0.05, ** *p* < 0.01.

**Figure 7:**
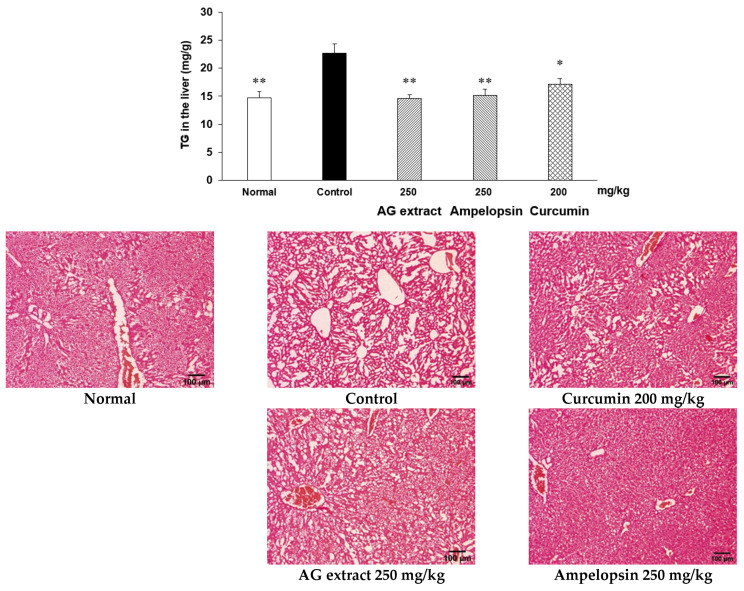
Effects of AG extract and ampelopsin on liver TG and microscopic images of hematoxylin-eosin staining. Each column represents the mean ± SE (*n* = 4–5). Data were assessed by a one-way analysis (ANOVA) of variance followed by Dunnett’s method. Asterisks denote significant differences from the control group at * *p* < 0.05, ** *p* < 0.01.

**Figure 8:**
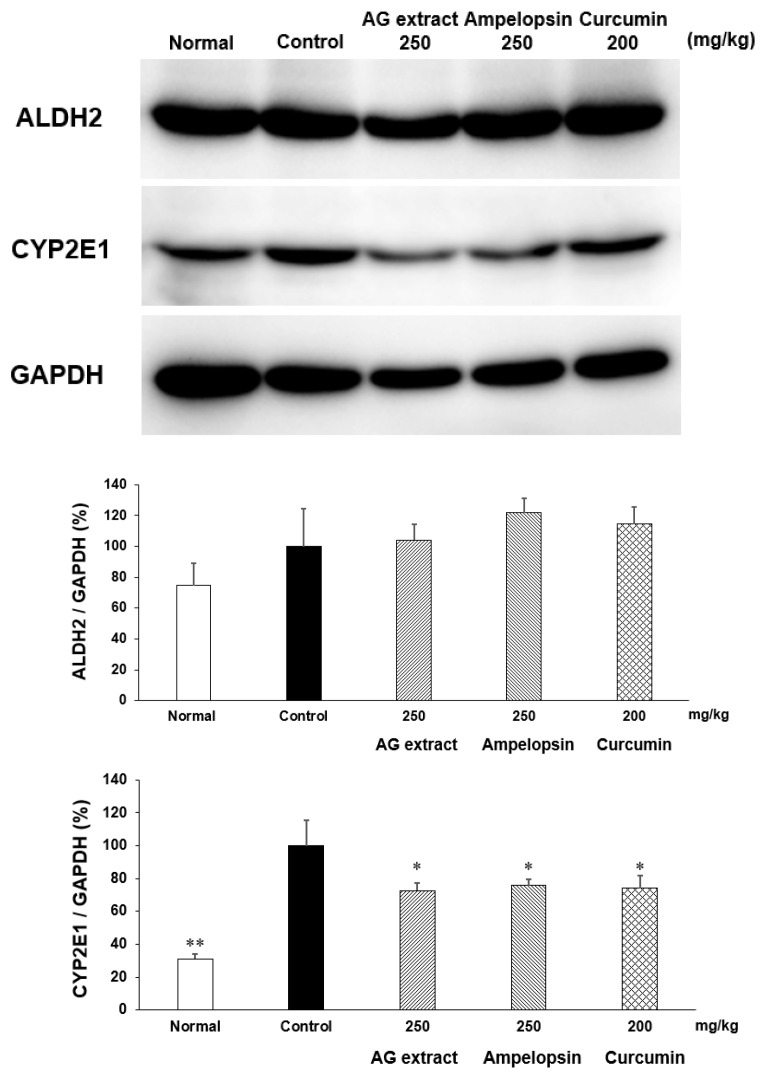
Effects of AG extract and ampelopsin on ALDH2 and CYP2E1 protein expression in the liver of alcohol diet-fed mice. Each column represents the mean ± SE (*n* = 3–4). Data were assessed by a one-way analysis (ANOVA) of variance followed by Dunnett’s method. Asterisks denote significant differences from the control group at * *p* < 0.05, ** *p* < 0.01.

In comparisons with the control group, the concentrations of ethanol and GOT in blood were slightly lower in the groups treated with AG extract (250 mg/kg), ampelopsin (250 mg/kg), and curcumin (250 mg/kg, Figure 6). Significant decreases were observed in GPT in the groups treated with ampelopsin and curcumin. The contents of TG in the liver were also significantly reduced in the groups treated with AG extract (250 mg/kg), ampelopsin (250 mg/kg), and curcumin (250 mg/kg, Figure 7). Aldehyde dehydrogenase 2 (ALDH2) plays a crucial role in maintaining low blood levels of acetaldehyde during alcohol oxidation. In comparisons with the control group, hepatic ALDH2 was elevated in the groups treated with AG extract (250 mg/kg), ampelopsin (250 mg/kg), and curcumin (200 mg/kg) (Figure 8). Cytochrome P450, family 2, subfamily e1 (CYP2E1) is regarded as the most important enzyme in the microsomal ethanol-oxidizing system. It has been shown to increase free radical and acetaldehyde production, which may deplete intracellular defenses against oxidative stress, further damaging hepatocytes [14]. In comparisons with the control group, a significant decrease in hepatic CYP2E1 was observed in the groups treated with AG extract (250 mg/kg), ampelopsin (250 mg/kg), and curcumin (200 mg/kg, Figure 8).

## 3. Discussion

**Figure 9:**
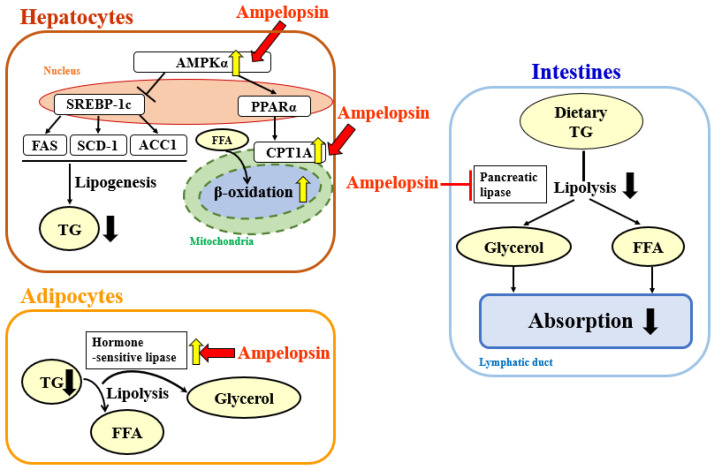
Potential underlying pathway by which AG extract and ampelopsin inhibits lipid absorption and fat accumulation and promotes fat decomposition.

In the present study on the anti-obesity effects of AG extract and ampelopsin in HFD-fed mice, AG extract (250 and 500 mg/kg) and ampelopsin (250 and 500 mg/kg) significantly suppressed increases in body weight, liver weight, and epididymal and perirenal fat volumes. Since abnormal lipid metabolism in the liver is always regarded as the main reason for obesity, many research groups have focused on lipid metabolism and its related mechanisms in the liver. Fang et al. reported that AMPKα inhibited the expression of sterol regulatory element binding protein 1c (SREBP-1c) and up-regulated the transcription levels of peroxisome proliferation-activated receptor α (PPARα), thereby affecting lipogenesis and lipid oxidation in the liver [15]. Shimoda et al. revealed that purple tea extract significantly suppressed increases in body weight and abdominal and liver fat accumulation associated with HFD-induced NAFLD in mice by up-regulating the expression of CPT1A in the liver [16]. CPT1 is a mitochondrial enzyme that is responsible for the β-oxidation of long-chain fatty acids. CPT1 includes three isozymes, with CPT1A being the most abundant in the liver. As shown in the summary of metabolic pathways in Figure 9 [11,15,16,17], we selected downstream CPT1A as the target protein and demonstrated that AG extract significantly enhanced CPT1A protein expression, which is consistent with previous findings [11]. In additional, Zeng et al. [18] reported that AG extract and ampelopsin inhibited the protein expression of SREBP-1c, fatty acid synthase (FAS), and ACC, which were responsible for lipogenesis in the liver; however, in comparisons with CPT1A, the effects of AG extract on these proteins appeared to be weaker. Also, another protein, acyl-coenzyme A oxidase 1 (ACOX1) was reported to involve in β-oxidation [19], we will verify it in future. Therefore here, AG extract and ampelopsin inhibit lipid accumulation and enhance lipid metabolism in vivo, possibly by suppressing lipogenesis and promoting lipid oxidation in the liver (Figure 9). The effects of AG extract and ampelopsin on serum TG levels in olive oil-loaded mice were investigated in in vivo experiments in the present study. Reductions in serum TG levels were observed in the groups treated with AG extract and ampelopsin (Figure 9). Therefore, AG extract and ampelopsin effectively suppressed lipid absorption.

The 3T3-L1 cell line has been used in many studies on adipogenesis and the biochemistry of adipocytes, and this cell line was originally developed by clonal expansion from murine Swiss 3T3 cells [20,21]. Since the suppressive effects of AG extract and ampelopsin on fat accumulation were confirmed in mice, we attempted to verify whether AG extract and ampelopsin exerted the same effects in cells. 3T3-L1 cells in medium containing insulin differentiate from pre-adipocytes into adipocytes, which continue to accumulate TG. In the present study, AG extract and ampelopsin significantly decreased the accumulation of TG in 3T3-L1 adipocytes after 48 h, particularly at concentrations of 10 and 30 µg/mL. Liu et al. previously reported that ampelopsin enhanced glucose metabolism and decreased adipogenesis by inhibiting the mitogen-activated protein kinase/extracellular signal-regulated kinase (MEK/ERK) pathway and down-regulating the phosphorylation of PPARγ in 3T3-L1 cells [22]. These findings are consistent with the present results. In the study by Liu, ampelopsin was added at the beginning of the differentiation of 3T3-L1 cells, and the findings obtained indicated that it suppressed the differentiation of adipocytes. In the present study, we assumed that AG extract and ampelopsin facilitated the decomposition of TG because TG had already accumulated in cells before these treatments. These results suggest that AG extract and ampelopsin not only suppress the formation of TG, but also promote their decomposition.

PPARs are a class of nuclear receptor proteins that play essential roles in the regulation of cellular differentiation, development, and metabolism [23,24]. Three isoforms of PPARs have been identified to date: α, β, and γ [25,26]. The α isoform is expressed in the liver, kidneys, heart, muscle, and adipose tissue upstream of CPT1A, and is closely associated with the inhibition of lipid absorption and accumulation by AG extract [11]. The β isoform is expressed in many tissues, but is prominent in the brain and skin, and plays roles in the oxidation of fatty acids and the energy balance [27]. The γ isoform exists in all tissues, is predominantly expressed in adipose tissue, and is essential for the differentiation and function of adipocytes [27,28]. A previous study reported that PPARγ was the most highly expressed and necessary in adipocytes for differentiation and is regarded as a master regulator of adipogenesis and a key regulator of gene expression in matured adipocytes [29,30,31]. Besides these, recently, lots of experiments related to obesity in whole-body PPARα^−^ deficient mice (PPARα^−/−^) and in mice lacking PPARα only in hepatocytes (PPARα^hep−/−^) were performed. For example, Regmier et al. [32] and Montagner et al. [33] reported that in PPARα deficient HFD mice, body weight, serum TG, GOT and GPT, liver inflammation, liver steatosis and other obesity related index would be significantly higher than control group, which indicated the importance of PPARα in lipid metabolism. Collectively, these findings indicate that PPARs contribute to the effects of AG extract and ampelopsin in 3T3-L1 cells. In the present study, we only confirmed the concentration of TG that accumulated, and, thus, further studies are needed to obtain insights into the underlying mechanisms. Besides cell experiments, two in vitro experiments, the rat epididymal fat pad test and pancreatic lipase activity test, were performed. In the rat epididymal fat pad test, the contents of FFA and glycerol released from fat pads were significantly increased, which suggested that AG extract and ampelopsin promote fat degradation and prevent abdominal fat accumulation (Figure 9). Gordon reported that PPARα could target uncoupling protein 1 (Ucp1) and adrenoreceptor β3 (Adrb3) [34], also in our results, AG extract and ampelopsin promoted TG decomposition in dose non-dependent which needed more experiments to verify the effect. Pancreatic lipase, also known as pancreatic triacylglycerol lipase or steapsin, is an enzyme that is secreted from the pancreas. It is a primary lipase enzyme that may hydrolyze dietary fat molecules into glycerol and FFA in the digestive system. The present results indicated that AG extract and ampelopsin significantly inhibit pancreatic lipase activity to reduce fat absorption (Figure 9).

**Figure 10:**
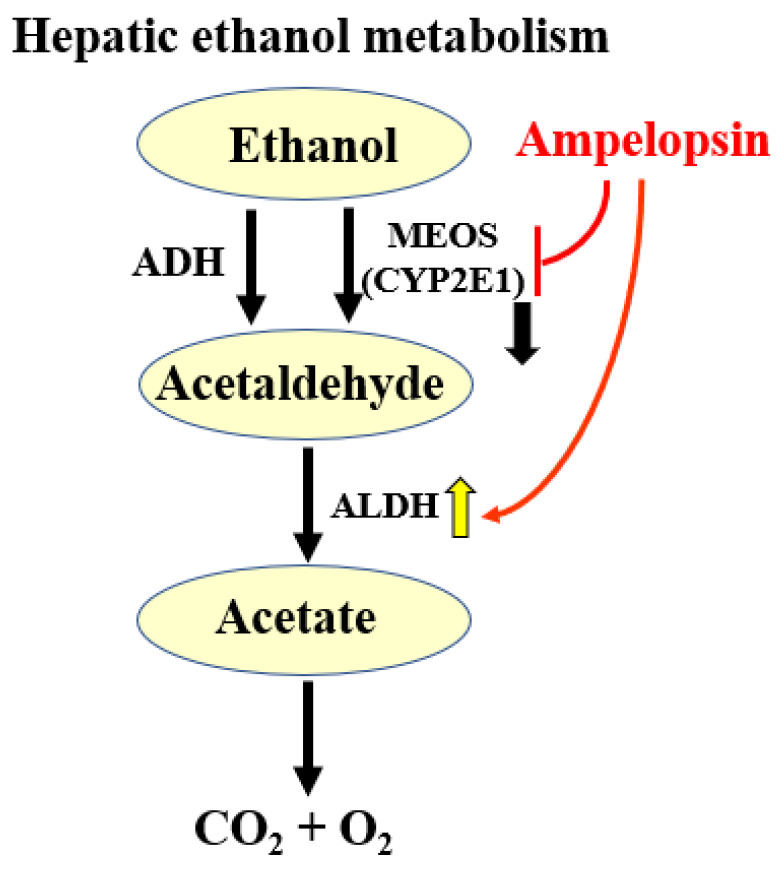
Potential underlying pathway by which AG extract and ampelopsin contribute to ethanol metabolism in the liver.

Collectively, the results of our in vivo and in vitro experiments indicated that in NAFLD, AG extract and ampelopsin effectively inhibited the absorption and accumulation of TG, while also promoting their decomposition. However, obesity induced by an excessive alcohol intake is also becoming a public health concern [35]. Indeed, excessive alcohol consumption is associated with a number of health issues, including alcohol-induced weight gain, which has been investigated for many years [36,37,38]. Therefore, a well-established ALD mouse model was used in the present study to assess the effects of AG extract and ampelopsin. The results obtained showed that AG extract and ampelopsin reduced the serum levels of ethanol, GOT, and GPT and liver TG, and modified the expression of ALDH and CYP2E1 in the liver. The slight decrease observed in serum ethanol suggested that AG extract and ampelopsin weakly promoted alcohol metabolism. The results of Western blot experiments provided supportive evidence, with a slight increase being noted in the expression of ALDH2, which plays a crucial role in alcohol oxidation. The main pathway for alcohol metabolism involves two enzymes, alcohol dehydrogenase (ADH) and ALDH. These two enzymes contribute to the degradation of alcohol and its elimination from the body. In the first step, ADH metabolizes ethanol to acetaldehyde, a highly toxic substance that is a known carcinogen. In the second step, acetaldehyde is metabolized by ALDH to less toxic acetate, which is further broken down into water and carbon dioxide for elimination (Figure 10) [39]. An excessive alcohol intake may activate the hepatic microsomal ethanol-oxidizing system (MEOS). CYP2E1 is considered to be the most important enzyme in MEOS. When ethanol is metabolized by CYP2E1, highly reactive oxygen-containing molecules or reactive oxygen species are produced. These free radicals deplete intracellular defenses against oxidative stress, further damaging hepatocyte proteins and DNA, or interact with other substances to create carcinogens [40]. In the present study, significant inhibitory effects on CYP2E1 were observed, which indicates the potential of AG extract and ampelopsin to protect the liver from alcohol-induced fatty liver more than promoting the metabolism of alcohol. A significant reduction in TG levels in the liver was also observed in the present study. However, in the study by Silva [13], significant effects on ALDH2 and CYP2E1 were detected. The administration of ampelopsin was via the intraperitoneal route instead of orally and the experimental term was 2 months. Since flavonoids always have weak bioavailability in vivo, they may have selected intraperitoneal injections as the administration route in addition to a long experimental term. These findings and the present results collectively suggest that AG extract and ampelopsin more effectively promoted alcohol metabolism, which we intend to investigate in future studies.

## 4. Materials and Methods

### 4.1. Materials and Preparation

AG extract (50% ampelopsin) and ampelopsin (98%) used in the present study were purchased from Hunan Nutramax Inc. (Hunan, China). In cell experiments, AG extract and ampelopsin were dissolved in dimethyl sulfoxide (DMSO). In animal experiments, AG extract and ampelopsin were suspended in a solution of 5% gum Arabic.

### 4.2. Animals and Cells

Animal experiments were performed in accordance with the Guidelines for Animal Experimentation (Japan Association for Laboratory Animal Science, 1987). All animal experiments were approved by the Ethics Committee of Oryza Oil & Fat Chemical Co., Ltd. (ORZ-T-001, 1 May 2020). Male ICR mice aged 6 and 9 weeks old, male C57BL/6 mice aged 6 weeks old, and SD rats aged 5 weeks old were purchased from Japan SLC, Inc. (Shizuoka, Japan) and accommodated for one week under a constant temperature (23 ± 2 °C) and illumination conditions (12 h day and night cycle) before experiments. Mice and rats were allowed access to food and water ad libitum. Standard CE-2 non-purified diet and HFD (HFD32) were purchased from Clea Japan, Inc. (Shizuoka, Japan), and Lieber-DeCarli alcohol diets were purchased from Research Diets, Inc. (New Brunswick, NJ, USA). Mouse 3T3-L1 cells were obtained from the JCRB Cell Bank (Osaka, Japan).

### 4.3. Chemicals and Reagents

T-Cho E TEST WAKO, TG E TEST WAKO, Dulbecco’s modified Eagle medium (DMEM), fetal bovine serum (FBS), phosphate-buffered saline (PBS), penicillin-streptomycin solution (PS), bovine serum albumin (BSA), DMSO, glucose, gum Arabic, skim milk, olive oil, and a FFA kit were purchased from FUJIFILM Wako Pure Chemical Industries Ltd. (Osaka, Japan). 1-Methyl-3-isobutylxanthine (IBMX), NA, and dexamethasone (DEX) were purchased from Sigma-Aldrich (St. Louis, MO, USA). A rabbit anti-CPT1A antibody and rabbit anti-CYP2E1 antibody were obtained from Abcam (Cambridge, UK). A rabbit anti-ALDH2 antibody was obtained from ProteinTech Group, Inc. (Rosemont, IL, USA). A mouse anti-glyceraldehyde-3-phosphate dehydrogenase (GAPDH) monoclonal antibody, radioimmunoprecipitation (RIPA) lysis and extraction buffer, protease and phosphatase inhibitor cocktail, and Pierce Western Blotting Substrate Plus were purchased from Thermo Fischer Scientific Inc. (Waltham, MA, USA). Horseradish peroxidase (HRP)-conjugated goat anti-rabbit IgG and anti-mouse IgG were purchased from Merck Millipore (Darmstadt, Germany). Insulin and orlistat (Xenical) were obtained from F. Hoffmann-La Roche, Ltd. (Basel, Switzerland). Laemmli sample buffer and polyvinylidene fluoride (PVDF) membranes were obtained from Bio-Rad Laboratories Inc. (Hercules, CA, USA). A glycerol kit was obtained from Cayman Chemical (Ann Arbor, MI, USA). F kit ethanol was obtained from R-Biopharm AG (Darmstadt, Germany). Lipase Kit S was purchased from DS Pharma Biomedical Co., Ltd. (Osaka, Japan). Methanol, acetonitrile, and DMSO for sample preparation and liquid chromatography were of HPLC or biochemical grade.

### 4.4. Detection of Ampelopsin in AG Extract

The HPLC system consisted of a Shimadzu (Kyoto, Japan) prominence liquid chromatograph pump, SPD-M20A prominence diode array detector, SIL-20AC prominence autosampler, CTO-20AC column oven, DGU-20A_5_ online degasser, and CBM-20A prominence communications bus module, and data were recorded using LC solution software (Version 1.25 SP5) (Shimadzu Co.).

A liquid chromatographic analysis was performed using a SHISEDO Capcell PAK C18 column (SG120, 250 mm × Φ4.6 mm, 5 μm, Osaka Soda, Osaka, Japan) and the column temperature was kept constant at 40 °C. The mobile phase was composed of a mixture of methanol, water with H_3_PO_4_ [41,42], mobile phase A was composed of 150 mL methanol, 850 mL water, and 10 mM H_3_PO_4_, and mobile phase B was composed of 700 mL methanol, 300 mL water, and 10 mM H_3_PO_4_. The following gradient program was used: the initial elution condition was A–B (100:0, *v*/*v*) from 0–10 min, changed to A–B (75:25, *v*/*v*) from 10–15 min and maintained from 15–25 min, changed to A–B (50:50, *v*/*v*) from 25–30 min and maintained from 30–40 min, changed to A–B (25:75, *v*/*v*) from 40–45 min and maintained from 45–55 min, changed to A–B (0:10, *v*/*v*) from 55–60 min and maintained from 60–70 min, and finally changed to A–B (100:0, *v*/*v*) from 70–80 min. The flow rate was 1.0 mL/min. The detector wavelength was set at 254 nm and the loading volume was 5 μL. All samples were dissolved by the mobile phase for the next detection.

### 4.5. HFD-Induced NAFLD in Mice

All mice (ICR aged 9 weeks) were randomly divided into six groups, the control and treatment groups of mice were fed with HFD32 for 14 days. For normal mice group, CE-2 diet was fed as the standard diet. AG extract (250, 500 mg/kg) and ampelopsin (250, 500 mg/kg) were administered orally to treatment groups once a day for 14 days. Then mice were anesthetized with isoflurane and blood was collected from abdominal ventral aorta, TG and T-Cho in serum were measured with the kit respectively. The liver, epidydimal and perirenal fats were removed and weighed. After weighing, the liver specimen was quickly stored at −80°C for the following Western blot experiments. To determine the CPT1A protein expression, the liver specimens were homogenized in RIPA extraction and isolation buffer containing protease and phosphatase inhibitor cocktail. The concentration of protein was adjusted with distilled water to 4 mg/mL. The lysate was mixed with the same volume of Laemmli sample buffer 65.8 mM Tris-HCl, 2.1% sodium dodecyl sulfate (SDS), 5% 2-mercaptoethanol, 26.3% glycerol and 0.01% bromophenol blue] and heated at 95 °C for 5 min. The heated solution (20 μg) was electrophoresed on 10% SDS gel. Separated protein was then transferred to a PVDF membrane. After blocking of the membrane with 5% skimmed milk, CPT1A expression was detected by rabbit anti-CPT1A antibody (1:10,000) and HRP-conjugated anti-rabbit IgG (1:25,000). GAPDH was detected by mouse anti-GAPDH antibody (1:10,000) and HRP-conjugated anti-mouse IgG (1:25,000). Detection was performed using by Pierce Western Blotting Substrate Plus and an imaging system (ImageQuant LAS500, Cytiva, Marlborough, MA, USA) [16,43].

### 4.6. Serum TG Changes in Olive Oil-Loaded Mice

The test method used was described in our previous study [43,44]. Mice (ICR aged 6 weeks) were fasted for 15 h and blood samples were collected from the orbital sinus under anesthesia using a glass capillary. Thirty minutes later, AG extract (250, 500 mg/kg), ampelopsin (250 mg/kg), and orlistat (20 mg/kg) were orally administered to mice. After 1 h, olive oil (5 mL/kg) was orally loaded and blood samples were collected 2, 4, and 6 h later. Blood samples were centrifuged (3000 rpm, 10 min) to obtain serum. TG levels were measured by a Wako TG E TEST kit.

### 4.7. TG Decomposition in Differentiated 3T3-L1 Adipocytes

The mouse embryonic fibroblast cell line, 3T3-L1 was maintained in DMEM (low glucose) containing 10% FBS and 1% PS solution at 37 °C under a 5% CO_2_ atmosphere. Adipocyte differentiation was induced as previously described [22,23,24]. Briefly, 3T3-L1 cells were seeded on 96-well plates (7 × 10^3^ cells/well) and 24-well plates (3.5 × 10^4^ cells/well). Two days after reaching confluence, the medium was changed to differentiation medium containing DMEM (high glucose), 10% FBS, 1% PS, 0.5 mmol/L IBMX, 0.25 μmol/L DEX, and 10 μg/mL insulin for 2 days. Cells were then maintained in DMEM (high glucose) containing 10% FBS, 1% PS, and 10 μg/mL insulin with medium replaced every 2 days for 6 days, until more than 80% pre-adipocytes had differentiated into adipocytes. AG extract and ampelopsin were then added and incubated for another 2 days. After sample treatment, MTT was added to the plates and incubated for another 4 h to evaluate cell viability. When the final incubation was complete, formazan products in the well were dissolved in 0.1 mL DMSO and the absorbance of the resulting solution was measured at 570 nm with a microplate reader. The percentage of viable cells was calculated as follows: Cell viability (%) = (A treated/A control) × 100. In 24-well plates, Oil Red O was used to stain and detect the concentration of TG that accumulated [22]. Briefly, cells were washed twice with PBS and fixed with 10% formaldehyde at room temperature for 10 min. Formaldehyde was then removed and washed twice with PBS. Oil Red O solution (0.3% in isopropanol diluted with water 3:2) was added and left for 20 min. Cells were washed with PBS and photographed using a Leica DMI6000 B microscope (Leica Microsystems, Wetzlar, Germany). The staining dye was dissolved with isopropanol (0.5 mL) and quantified by a microplate reader at a wavelength of 540 nm.

### 4.8. FFA and Glycerol Release from Rat Epididymal Fat Pads

Epididymal adipose tissues from rats (SD rats aged 5 weeks) were divided into 200 mg sections, cut into small pieces of 1~2 mm, and then placed in a 12-well plate with Krebs-Ringer buffer (including 2% BSA and 5 mmol/L glucose). After 5 min, AG extract and ampelopsin were added. NA (10 μg/mL) was added as a positive group. Plates were incubated at 37 °C with gentle shaking for 60 to 90 min, solutions were centrifuged (15,000 rpm, 10 min) to obtain supernatants, and FFA and glycerol in the solution were then assessed according to the instructions of the kit [45,46,47].

### 4.9. Pancreatic Lipase Inhibition Test

This test was performed according to the procedure described in the kit. Coloring agent, pancreatic lipase, an esterase inhibitor, and AG extract and ampelopsin dissolved in DMSO were added to a test tube and mixed. After mixing, each test tube was preheated at 30 °C for 5 min. The substrate solution was added to the AG extract and ampelopsin test tubes and mixed. The mixtures were then incubated at 30 °C for 30 min. The stopping reaction was added to each test tube and mixed. The substrate solution was subsequently added to the blank test tube and mixed. The absorbance of the solution in each test tube was measured at a wavelength of 412 nm [48,49].

The lipase inhibition ratio was calculated as follows:

Lipase inhibition ratio (%) = [1 − (S − Sb/C − Cb)] × 100

where S: the absorbance of each AG sample, Sb: the absorbance of the blank (AG sample), C: the absorbance of the control, Cb: the absorbance of the blank (control). IC_50_ values were determined graphically by drawing a concentration-inhibition curve.

### 4.10. Alcohol Diet-Induced Alcoholic Fatty Liver Disease in Mice

All mice (C57BL/6 mice aged 6 weeks) were randomly divided into five groups; the normal group was fed the Lieber-DeCarli diet without alcohol, while the four other groups were fed Lieber-DeCarli alcohol diets plus one binge [50]. AG extract (250 mg/kg), ampelopsin (250 mg/kg), and curcumin (200 mg/kg) were orally administered to treatment groups once a day for 11 days. Mice were then anesthetized with isoflurane, blood was collected from the abdominal ventral aorta, and the serum levels of ethanol, GOT, and GPT were measured with the kit. Livers were removed and quickly stored at −80 °C for subsequent assessments of TG and Western blot analyses. To measure TG levels, specimens (approx. 50 mg) were homogenized in a mixture of chloroform and methanol (2:1) and centrifuged (3000 rpm, 10 min) to obtain the supernatant. The solvent was evaporated by N_2_ gas and H_2_O (0.3 mL) was added to disperse the homogenate. TG contents were measured using a Wako Triglyceride (TG) E TEST kit. To assess ALDH2 and CYP2E1 protein expression, liver specimens were homogenized in RIPA extraction and isolation buffer containing protease and phosphatase inhibitor cocktail. Electrophoresis and membrane transfer were performed using the methods described above (Section 4.5). After blocking of the membrane with 5% skim milk, ALDH2 and CYP2E1 expression was detected by rabbit anti-ALDH2 (1:3000), the CYP2E1 antibody (1:5000), and HRP-conjugated anti-rabbit IgG (1:25,000). GAPDH was detected by the mouse anti-GAPDH antibody (1:10,000) and HRP-conjugated anti-mouse IgG (1:25,000). Detection was performed using the imaging system described above (Section 4.5). Liver slices were also photographed using a microscope as described above (Section 4.7).

### 4.11. Statistical Analysis

Data were expressed as means ± standard errors (SE). Significant differences between groups were assessed by a one-way analysis (ANOVA) of variance followed by Dunnett’s method. Values of *p* < 0.05 or *p* < 0.01 were considered to be significant.

## 5. Conclusions

In the present study, AG extract and its main active ingredient ampelopsin exerted inhibitory effects on the absorption and accumulation of TG, while AG extract and ampelopsin also showed good effects on TG decomposition both in vivo and in vitro experiments. All these results indicate the potential of AG extract and ampelopsin as an excellent future functional food material, however, further studies are warranted.

## Figures and Tables

**Table 1:** Effects of AG extract and ampelopsin on body weights and weights of liver, epididymal and perirenal fat in HFD-fed mice.

	Dose (mg/kg)	Body Weight (g)	Liver (g)	Epididymal Fat (g)	Perirenal Fat (g)
Normal	-	45.24 ± 0.62 **	1.65 ± 0.04 **	0.98 ± 0.09 **	0.53 ± 0.07 **
Control	-	53.76 ± 0.81	2.15 ± 0.08	2.65 ± 0.18	1.05 ± 0.06
AG extract	250	47.85 ± 0.82 *	1.83 ± 0.04 *	1.88 ± 0.15 **	0.71 ± 0.05 *
	500	48.82 ± 1.97	1.85 ± 0.09 *	1.86 ± 0.16 **	0.85 ± 0.07
Ampelopsin	250	49.12 ± 1.94	1.80 ± 0.08 **	1.87 ± 0.16 **	0.84 ± 0.09
	500	48.54 ± 1.28	1.90 ± 0.06	1.87 ± 0.12 **	1.01 ± 0.11

Each value represents the mean ± SE (*n* = 7). Data were assessed by a one-way analysis (ANOVA) of variance followed by Dunnett’s method. Asterisks denote significant differences from the control group at * *p* < 0.05, ** *p* < 0.01.

**Table 2:** Effects of AG extract and ampelopsin on serum TG and T-Cho levels in HFD-fed mice.

	Dose (mg/kg)	TG (mg/dL)	T-Cho (mg/dL)
Normal	-	97.5 ± 7.3 *	148.7 ± 19.3 *
Control	-	144.9 ± 13.2	235.4 ± 36.2
AG extract	250	98.5 ± 12.1 *	199.1 ± 19.0
	500	106.0 ± 14.2	197.6 ± 11.6
Ampelopsin	250	87.8 ± 9.3 **	213.7 ± 10.5
	500	109.0 ± 9.8	214.7 ± 13.5

Each value represents the mean ± SE (*n* = 7). Data were assessed by a one-way analysis (ANOVA) of variance followed by Dunnett’s method. Asterisks denote significant differences from the control group at * *p* < 0.05, ** *p* < 0.01.

**Table 3:** TG contents and viability of 3T3-L1 cells treated with AG extract and ampelopsin.

			Concentration (µg/mL)				
		Control	1	3	10	30	100
TG (%)	AG extract	100.0 ± 1.1	90.1 ± 2.5	89.9 ± 2.2 **	85.6 ± 1.2 **	92.7 ± 2.1 *	85.2 ± 1.2 **
	Ampelopsin	100.0 ± 3.2	99.7 ± 2.1	90.0 ± 4.2	99.2 ± 6.0	91.7 ± 4.3	83.2 ± 1.7 *
Viability (%)	AG extract	100.0 ± 1.6	103.0 ± 1.0	100.0 ± 1.1	99.0 ± 1.5	99.4 ± 1.7	94.0 ± 1.4 *
	Ampelopsin	100.0 ± 2.4	99.7 ± 1.8	99.8 ± 2.8	101.8 ± 2.4	102.5 ± 2.4	89.5 ± 1.6 *

Each TG value represents the mean ± SE (*n* = 3–4), each viability value represents the mean with the SE (*n* = 5–6). Data were assessed by a one-way analysis (ANOVA) of variance followed by Dunnett’s method. Asterisks denote significant differences from the control group at * *p* < 0.05, ** *p* < 0.01.

**Table 4:** Lipolytic effects of AG extract and ampelopsin on the rat epididymal fat pad.

			Concentration (µg/mL)					
		Control	NA (10)	1	3	10	30	100
FFA (%)	AG extract	100.0 ± 0.3	478.4 ± 7.5 **	133.4 ± 1.4 **	137.4 ± 0.7 **	138.0 ± 0.3 **	136.4 ± 1.2 **	124.3 ± 0.3 **
	Ampelopsin	100.0 ± 1.4	559.1 ± 1.0 **	119.7 ± 1.0 **	117.0 ± 1.3 **	118.1 ± 1.8 **	123.6 ± 3.2 **	116.6 ± 0.4 **
Glycerol (%)	AG extract	100.0 ± 3.3	217.5 ± 2.8 **	117.1 ± 1.1 **	112.3 ± 0.4 **	119.3 ± 0.6 **	110.0 ± 0.9 *	102.2 ± 3.0
	Ampelopsin	100.0 ± 1.0	348.8 ± 3.0 **	108.3 ± 0.8 **	112.2 ± 1.8 **	106.8 ± 0.8 **	124.9 ± 2.0 **	95.6 ± 0.5

Each value represents the mean ± SE (*n* = 3). Data were assessed by a one-way analysis (ANOVA) of variance followed by Dunnett’s method. Asterisks denote significant differences from the control group at * *p* < 0.05, ** *p* < 0.01.

**Table 5:** Inhibition of pancreatic lipase activity by AG extract and ampelopsin.

	Inhibition (%)						
	Control	1 (µg/mL)	3	10	30	100	IC_50_ (µg/mL)
AG extract	0.0 ± 2.0	−0.5 ± 2.8	8.4 ± 0.7	22.8 ± 11.5	36.7 ± 2.4 **	58.8 ± 0.9 **	97.7
Ampelopsin	0.0 ± 3.9	17.3 ± 3.0	11.6 ± 3.8	28.8 ± 2.5 **	44.0 ± 2.3 **	66.7 ± 5.0 **	45.9
Orlistat	0.0 ± 3.8	77.4 ± 5.0 **	81.6 ± 1.5 **	90.2 ± 0.4 **	95.6 ± 0.1 **	97.4 ± 0.2 **	<0.5

Each value represents the mean ± SE (*n* = 3). Data were assessed by a one-way analysis (ANOVA) of variance followed by Dunnett’s method. Asterisks denote significant differences from the control group at ** *p* < 0.01.

## Data Availability

Data related to the present study are available from the corresponding author upon reasonable request.

## Sample Availability

Samples of the compounds (Ampelopsin or AG extract) are available from the authors.

## Abbreviations

AG	*Ampelopsis grossedentata*
NAFLD	non-alcoholic fatty liver disease
HFD	high-fat diet
CPT	carnitine palmitoyltransferase
GOT	glutamic oxaloacetic transaminase
GPT	glutamic pyruvic transaminase
TG	triglyceride
ALDH	aldehyde dehydrogenase
CYP2E1	cytochrome P450, family 2, subfamily e1
ACC	acetyl CoA carboxylase
FFA	free fatty acid
ADH	alcohol dehydrogenase
AMPK	adenosine 5′-monophosphate-activated protein kinase
SREBP-1c	sterol regulatory element binding protein 1c
PPAR	peroxisome proliferation-activated receptor
FAS	fatty acid synthase
ACOX1	acyl-coenzyme A oxidase 1
Ucp1	uncoupling protein 1
Adrb3	adrenoreceptor β3
MEK/ERK	mitogen-activated protein kinase /extracellular signal-regulated kinase
MEOS	microsomal ethanol-oxidizing system
ROS	reactive oxygen species
DMSO	dimethyl sulfoxide
T-Cho	total cholesterol
DMEM	Dulbecco’s modified Eagle medium
FBS	fetal bovine serum
PBS	phosphate-buffered saline
PS	penicillin-streptomycin solution
BSA	bovine serum albumin
IBMX	1-methyl-3-isobutylxanthine
NA	norepinephrine
DEX	dexamethasone
RIPA	radioimmunoprecipitation
HRP	horse radish peroxidase
PVDF	polyvinylidene fluoride
GAPDH	glyceraldehyde-3-phosphate dehydrogenase
HPLC	high performance liquid chromatography
